# A GERIATRIC EDUCATION COURSE SPANNING COMMUNITY AND HEALTH CARE ENVIRONMENTS

**DOI:** 10.1093/geroni/igad104.2357

**Published:** 2023-12-21

**Authors:** Michele Gareri, Susan Hazelett, Denise Kropp, Margaret Sanders, Jennifer Drost, Lori Kidd, Susan Fosnight

**Affiliations:** Summa Health , Akron, Ohio, United States; Summa Health Systems, Akron, Ohio, United States; Northeast Ohio Medical University, Rootstown, Ohio, United States; Northeast Ohio Medical University, Rootstown, Ohio, United States; Summa Health Systems, Akron, Ohio, United States; University of Akron, Akron, Ohio, United States; Summa Health System, Akron, Ohio, United States

## Abstract

To address gaps in the geriatrics-trained workforce, we implemented a Geriatric Resource Certificate (GRC) for interprofessional learners from healthcare and community provider settings. This program combines online self-paced didactics, real-time virtual lectures, and a real-world virtual team care planning experience. The education incorporates the 4Ms of the Age Friendly Framework and spans the geriatric experience from healthy aging through end-of-life. It includes Dementia Friends and ECHO sessions. The goal is to training people from a variety of care settings to be resource people regarding geriatric issues, much like the NICHE program in acute care. We had 37 learners who completed evaluations. Practice settings included ACO, Hospice, Emergency Department, nursing homes, hospitals, primary care, Area Agencies on Aging, and other community providers. Overall, the online didactics were rated an average of 4.53 on a 5 point Likert scale and the real-time lectures were rated an average of 4.41. The average score for “the GRC will help me provide care over the continuum” was 4.59. The average for the real team was 4.07, 4.26 for the ECHO, and 4.45 for Dementia Friends. All learners indicated they would refer others to the GRC. These results indicate that the GRC is an effective method to impart basic geriatric training into communities and raise awareness of resources available to assist older adults in maintaining their independence. In this era of high healthcare workforce turnover and staffing shortages, improving geriatric knowledge by training on-site geriatric resource staff is one way to improve care for older adults.

